# Advancing taxonomy and bioinventories with DNA barcodes

**DOI:** 10.1098/rstb.2015.0339

**Published:** 2016-09-05

**Authors:** Scott E. Miller, Axel Hausmann, Winnie Hallwachs, Daniel H. Janzen

**Affiliations:** 1 National Museum of Natural History, Smithsonian Institution, PO Box 37012, Washington, DC 20013-7012, USA; 2 SNSB—Zoologische Staatssammlung München, Münchhausenstraße 21, 81247, München, Germany; 3 Department of Biology, University of Pennsylvania, Philadelphia, PA 19104, USA

**Keywords:** DNA barcoding, cytochrome c oxidase I, biodiversity, interim taxonomy, Lepidoptera

## Abstract

We use three examples—field and ecology-based inventories in Costa Rica and Papua New Guinea and a museum and taxonomic-based inventory of the moth family Geometridae—to demonstrate the use of DNA barcoding (a short sequence of the mitochondrial COI gene) in biodiversity inventories, from facilitating workflows of identification of freshly collected specimens from the field, to describing the overall diversity of megadiverse taxa from museum collections, and most importantly linking the fresh specimens, the general museum collections and historic type specimens. The process also flushes out unexpected sibling species hiding under long-applied scientific names, thereby clarifying and parsing previously mixed collateral data. The Barcode of Life Database has matured to an essential interactive platform for the multi-authored and multi-process collaboration. The BIN system of creating and tracking DNA sequence-based clusters as proxies for species has become a powerful way around some parts of the ‘taxonomic impediment’, especially in entomology, by providing fast but testable and tractable species hypotheses, tools for visualizing the distribution of those in time and space and an interim naming system for communication.

This article is part of the themed issue ‘From DNA barcodes to biomes’.

## 1. Introduction

Much has been written on the importance of conserving the world's biodiversity, and the importance of understanding that diversity by expanding taxonomic knowledge of it, most recently highlighted by Wilson's [2] proposal to increase the area of natural reserves to half the surface of the Earth, and to accelerate the taxonomic inventory of the Earth's species. We have spent most of our careers working on biodiversity inventories, all kinds of which have now been dramatically changed through addition of the DNA barcoding tool for identifying, discovering and characterizing species, and communicating about them to all users across society. Here, we highlight three ongoing inventories to illustrate how DNA barcoding is contributing to the renaissance of taxonomy [3], while also linking species concepts to their ecology. Each essay represents not only the unique history and context of each project, but they also show the transformational nature of DNA barcoding.

## 2. The view from the field: Area de Conservacion Guanacaste, northwestern Costa Rica

The hope and expectation is that society will accept wild biodiversity as a legitimate co-occupant of the planet. Bioinventory starts in nature and arrives to society's brains and actions. During this process, a plethora of trials and tribulations accumulate before we can say, for example, with total confidence: ‘Oh, *Opsiphanes jacobsorum,* a large butterfly of Costa Rican mid-elevation intact rainforest, has just been discovered and today is a rare member of the fauna of Area de Conservacion Guanacaste (ACG) in northwestern Costa Rica’ [4]. For 150 years prior to the injection of DNA barcoding into the ACG Lepidoptera bioinventory [5], this rainforest species was confused with the dirt-common Mexico-to-Panama palm-eating *Opsiphanes fabricii* (still widely identified as *O. cassina*), a confusion discovered and eliminated by DNA barcoding of these two species by the ACG bioinventory 7 years before examination by any taxonomist, and substantiated by later-discovered morphology and behaviour.

The bioinventory of ACG caterpillars began in 1978 and ran for 25 years, just as it would have been conducted by any Victorian naturalist. Additionally, the adult resulting from each wild-caught (and often photographed) caterpillar was individually databased on-site, and multiple uniquely coded vouchers were preserved, largely owing to the impossibility of otherwise identifying their caterpillars in the absence of field guides, library, and accompanying taxonomists for this largely unknown tropical fauna then estimated at 9500 species of Lepidoptera.

However, in 2003 all that changed when Paul Hebert presented the concept of adding DNA barcoding for specimen identification and species discovery to the taxasphere's tool box [6], and we then offered him a leg from each of the 25 years of oven-dried and museum-spread ACG vouchers to test-drive the concept. The first example revealed 10 sympatric species of subtly variable and nearly identical showy and well known, frequently photographed and collected ACG adult skipper butterflies hiding inside one scientific name used for 225 years, a complex that then sorted cleanly against caterpillar colour patterns and food plants [7]. This routine has since been repeated with thousands of species in the ACG bioinventory, with the current record being 39 species of highly host-specific parasitic wasps that had been hiding under one morphology-based generalist name; these have subsequently been found to be distinguishable by minute overlooked differences discovered by a scrutinizing taxonomist guided by the barcodes and host records [8].

Continued barcoding, coupled with morphology (including colour patterns), natural history and within-ACG microgeography and ecosystem structure, have now raised the estimate to 15 000 species of Lepidoptera in an area the size of New York and its suburbs, containing three major tropical terrestrial ecosystems—dry forest, cloud forest and rain forest—and their intergrades. In practical terms, within weeks to months of when there is an oven-dried adult voucher, a leg in a lysis plate is couriered to the Biodiversity Institute of Ontario (BIO), just as we used to send our rolls of K25 film to Kodak. Within one to six months, there is a barcode with its within-BOLD (The Barcode of Life Database) massive out-bound and in-house comparability to join with what the bioinventory is telling us about food plant, appearance and micro-within-ACG location, and simultaneously to join with what other biodiversity users have in mind. Some of the major DNA barcoding-facilitated processes in the tortured travelogue from the forest to someone's brain are:

(1) When the parataxonomists find a caterpillar on its food plant or select an adult from a light trap, at that time its on-site database record receives all the collateral available, including best-guess interim species and higher taxon names. Along its early journey that name may be upgraded by a handler, but after several months the BOLD-generated and inventorier-screened name is applied to the record. No taxonomist has had to directly bother with this process or specimen to this point, though the parataxonomists and processors along this trip all frequently logic-check the interim identification. The addition of a ‘better’ name creates a pulse of confirmations and explorations within the bioinventory if there is discordance between the database collateral and the previous biological collateral for that name. In the meantime, the specimen is bumping along its journey to a final home in a permanent public institutional collection (for ACG barcoded Lepidoptera, this is usually the Smithsonian National Museum of Natural History or Costa Rica's Museo Nacional collection (formerly INBio collection); for parasitoids, it is the institutional collection where the collaborating taxonomist for that higher taxon resides).
(2) However, the barcodes may indicate that the specimen could be one of several species, described or otherwise. Then, before the specimen enters its ‘final’ home, it lands on the desk of a participating taxonomist to be puzzled at further as to whether the seemingly applicable name truly encompasses more than one ‘thing’. All collateral information, including the barcodes and their comparison with those of other specimens and species in BOLD, come into play again. A barcode multichotomy that is more than trivial wobble in a specimen-rich cluster of full-length barcodes in a Neighbour-Joining (NJ) tree, and its accompanying collateral, may suggest that (i) it is more than one species, (ii) one haplotype is the true barcode and the other a pseudogene (e.g. all of one side of split is one sex and the other side approximates a 50 : 50 sex ratio), (iii) the sample has been contaminated by another genome, (iv) a book-keeping error has occurred, (v) some portion of the collateral was incorrect (e.g. caterpillar food plant misidentification, location synonymy), or (vi) there is a true barcode polymorphism. After barcoding more than 11 000 species of ACG Lepidoptera (and 2000+ species of parasitoid Hymenoptera and Tachinidae), the inventory has experienced multiple cases of all six but the latter has been substantiated only rarely; however, recall that ACG is a small area of only 120 000 terrestrial hectares and detailed sequence examination is both costly and disruptive to the mass flow of specimens. Often resolution of possible cryptic species is better achieved by increasing the sample size and intense scrutiny of specimens and their collateral, and by being suspicious of shallow emerging dichotomies in an NJ tree, than with expensive re-sequencing. Without the extremely reliable and taxonomist-independent DNA barcode to guide the inventory, both in real time and by integration with previously inventoried vouchers in the on-site databases and Internet-accessible BOLD, we would be operating at a far lower level of species-level resolution, with species complexes and batches of ordinary look-alikes being viewed as single species, if even detected. And commonly the field inventory has presumably reliable collateral (behaviour, food plants, seasonality, microgeography) to guide NJ tree interpretation, while the taxonomist is generally forced to act as though nature begins in the museum drawer.
(3) When the barcodes and collateral suggest that there are more species than meet a quick inspection, the field action tries to focus on getting more specimens of that complex, becoming suspicious of what were believed to be feeding or microgeographic ‘outliers’, and increasing efforts to see whether there are other similar species displaying the same class of multichotomy. For example, bioinventory barcoding has flushed out nearly ‘morphologically identical’ pairs occupying (i) the upper and lower slopes of a 1500 m volcano, (ii) dry forest and immediately adjacent rainforest, (iii) one ecosystem and its adjacent intergrade, (iv) drastically different food plant families, and (v) the crowns of adult trees and conspecific low saplings. On the other hand, we have species that have long been recognized by standard morphology-based taxonomy, yet differ by only 1–2 base pairs of a 658 base pair DNA barcode; a barcode split is a split, and the more sample and the more collateral substantiation, the more convincingly they are treated as two (or more) species [9].
(4) A barcode-confirmed species or complex gives the data-gatherers confidence that what they are seeing is real. That the two identical *Prepona* caterpillars (and their adults) feeding side-by-side on Fabaceae and Chrysobalanaceae, two very different plant families, really are two species (as original museum-based taxonomists once described them, only to have later museum-based taxonomists pool them under one name), allows the inventory to be confident in the results of its parasitoid component for these two species, even though no specimen of parasitoid host is barcoded. That the minute differences in colour pattern of two species of *Phoebis* whose caterpillars eat the same *Inga* in the same place at the same time [9] signal two quite different species, again long ago separated by museum taxonomists and then more recently erroneously pooled, is only believed when one sees the barcode data for them. In this progression, it is unavoidable that the inventory declares, for example, three species of common butterflies to be different when they are separable (at present) by only their barcodes and some non-morphological collateral [10].
(5) The ever-climbing species-per-specimen-collected curve, based on barcoded masses of thousands of small bark-coloured micro moths from light traps, is the only stimulus there can ever be to keep inventorying what appear to be (but are not) the same species repeatedly across all ACG ecosystems and seasons. Yes, this means that the museum is the recipient of thousands of undescribed species, as was emphatically complained at by a taxonomist reviewer of an NSF grant proposal. But is the solution to only put described species into museums? Of course not. Any museum and taxonomist receiving the specimens already sorted by DNA barcode and along with the collateral in the specimen records can aim surgically at the problems and a few individuals of each species revealed by barcoding. For example, to inventory and organize the thousands of specimens of phycitine Pyralidae of hundreds of ACG sympatric species by dissection will drown any taxonomist. However, if they arrive as barcoded, and therefore as packets of specimens with interim names, the taxonomist can surgically assign scarce time, space and cash resources, as well as ask for more of this or that while the inventory is still in motion. And even before they enter a taxonomist processing mill, the specimens and data can be compared across geography and taxonomies through the BOLD intermediary—which not only applies names already worked out elsewhere, but also reveals unintentionally incorrect synonymies in the ACG and other inventories (where it is commonplace for the same name to be applied accidentally to quite obviously different species by different projects in other parts of the tropics). Finally, via their barcodes, these as yet unidentified specimens can be queried via the Internet and further compared by other taxonomic or economic projects, a frequent occurrence with the ACG inventory (e.g. is a south Texas rare species really the same as its look-alike found in the ACG dry forest, one of which has a name and the other not?). But there is the barcoded-inventory-created snarl: if one named species dissolves into three, not only is there the question of which of the three actually matches the holotype, but also if that one species can dissolve into three in ACG, how many species overall are included in what was thought to be the range of one species from south Texas to Panama or Argentina? The barcoded field inventory abruptly highlights the desire for museums to house and study far more specimens of ‘a species’ over its geographical range than seemed necessary before.
(6) The continued and continuous barcoding of the specimens reared and light-collected has led to the group of barcoded specimens that have a scientific name or interim name, whether synonymous with a BIN or with a portion of a BIN, becoming pragmatically ‘the ACG species’. The name applied is yet another portion of collateral attached to that set of *N* specimens representing a hypothesized biological species for a wide variety of analyses. When a pulse of several thousand inventory barcodes of many families is received by BOLD, they reside there until the inventory requests an NJ tree for a particular family. That tree containing as many as 5–50 k barcodes may then be found to have 300, for example, that the inventory had assigned a simple interim placeholder, such as hespJanzen01 Janzen01. This general interim ‘name’ is not italicized and represents perhaps 35 species of hesperiine Hesperiidae. It is then only a matter of an hour to skim down that NJ tree and assign the name already assigned to a barcode group (BIN or otherwise) to the new specimen falling into that group, or realize that the group is breaking into more than one by the addition of these new samples. The accompanying Excel file is then uploaded to BOLD. The same process of identification of those 300 specimens would have taken a museum-based taxonomist several days of intense scrutiny and dissections, rich in doubts and impossibilities, even if there were a taxonomist in the field or other way-station to do it (and with what tools?). After years of this process, the same specimens then arrive at their final museum destination already sorted to species and with any available names already applied to them because select nameable and barcode group-representative specimens in the meantime have been sent as images or actual specimens to a collaborating taxonomist. By this method, for example, 20 000+ specimens of 600+ species-sorted Hesperiidae have been deposited in the Smithsonian, and it is clear which species/specimens are undescribed and need more taxonomic treatment. This process is working well for all 11 000+ species of Lepidoptera found to date by the ACG bioinventory, but obviously requires parataxonomists + transit protocols + taxasphere.
(7) If each individual parataxonomist had their own personal on-site barcorder, the field inventory would become yet much more surgical and use its scarce time and resources more effectively, as would the receiving taxasphere. Specimens would lose a piece of a leg to the barcorder, and the inventory would instantly know if it was worthy of more investment, something that today is only possible after the specimens have made their way through the entire processing and analysis stage, and a barcode is returned via BOLD. Species complexes would be revealed near the time of their first collection, which is always the best time to attempt to get more of them to clarify taxonomic analyses later and understand their field biology at any time. Furthermore, by their very nature, it is impossible for a massive reference collection, published hard copy guides or working taxonomists to be on-site at the time of the field inventory and guide the same process of yes/no more sampling. A personal identification process in place will also allow connection to biodiversity across all of society, quite aside from the dedicated effort to understand what is inside the protected wild places called national parks. On the other hand, if the specimens are barcode-identified at the time of capture, the vast majority of them will be discarded. By not making their way to museums or other storage depositories, they are thereby not entering into all the future uses for which museum specimens are retained (e.g. every bug on a pin or in a deep freeze is a time-stamped genome). We cannot lose sight of the fact that the purpose of the field inventory is both to know what is where and what it does, and to generate comparative information beyond the inventory.

## 3. The view from the museum: global inventory of the megadiverse Geometridae moths

Geometridae (looper caterpillars) are among the largest families in the animal kingdom, with some 23 000 currently described species [11,12], including many economically important species [13,14], and thousands more yet to be described. They are frequently a model taxon for ecological studies [15,16] and can be indicators for monitoring environmental changes [17].

**Figure 1.**
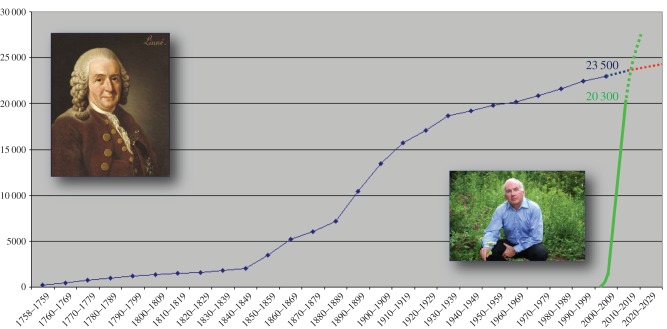
Carolus Linnaeus (left), the initiator of the ‘Linnean age of taxonomy’, with the morphological-descriptive assessment of some 23 000 geometrid species, achieved in 257 years. Paul D. N. Hebert (right), the initiator of the ‘Hebertian age of taxonomy’, has led to the molecular definition of some 20 000 geometrid BINs (approximately corresponding to species [18]) in just 12 years.

Despite great achievements in geometrid taxonomy in the past two and a half centuries, much remains to be done. Although increasing extinction rates beg for accelerated biodiversity assessment, the current rates of species description are low owing to (i) the lack of financial support for taxonomists, (ii) the poor quality of many historic descriptions, (iii) inaccessibility of relevant journals, and (iv) type specimens being accessible only by expensive travel to many different museums. In total, 19 000 out of 23 000 geometrid species descriptions (83%) were published before 1950 (figure 1), usually with very short diagnoses and poor or no images of critical characters. More than 8000 descriptions (43%) lack illustrations and several thousand are accompanied by low-quality paintings.

While 250 years of ‘traditional‘ morpho-analytical taxonomy have yielded the formal description of 23 000 geometrid species (figure 1), the real number of geometrid species on the Earth can be estimated to even much more than 40 000, when considering the ratio of described versus undescribed species in collections and the species complexes revealed by DNA barcoding (as represented by BINs on BOLD). Extrapolation from the mean species description rate of the last 50 years (70 species annually) shows that at least 230 years will be needed to document global geometrid biodiversity through ‘business as usual’. However, 300–400 years may be a more realistic time frame owing to a decrease in description rates as the end is approached.

For a change of pace, in just 12 years of DNA barcoding globally available museum geometrids, sequences belonging to more than 20 000 BINs were generated (approximately corresponding to species [18]; figure 1), thus making Geometridae by far the largest and best-covered family in BOLD.

The great success and rapid progress of the geometrid campaign was made possible through national/regional campaigns and focused taxon-sampling in museums. While 100 613 (72%) of all 138 810 barcoded geometrid individuals were younger than 5 years at the time of being processed, the remaining 28%—mostly taken from older special collections of major natural history museums—contributed to 15 067 BINs (77%). Major contributions to the geometrid campaign came from the Bavarian State Collection of Zoology, Munich (10 558 BINs/35 905 barcodes), Biodiversity Institute Ontario, Guelph (2287/32 564), Area de Conservacion Guanacaste (1700/17 000), Research Collection of Gunnar Brehm (2033/4965), Australian National Insect Collection, Canberra (1453/4631), and Smithsonian Institution, Washington (1412/4480). Large national or regional campaigns and total inventory approaches provided considerable amounts of geometrid data as well: Canada (549 BINs/22 207 barcodes), Australia (2006/19 503), Costa Rica (1700/17 000), USA (1319/13 975), Ecuador (2253/6684), Papua New Guinea (882/4022) and Germany (429/3998).

Many experts are involved in the subsequent taxonomic processing of barcode data in BOLD and specimen collateral (locality, morphology, dates and geography). This is complemented by DNA barcoding of specimens from expert collections (e.g. more than 5000 BINs from the Herbulot collection) and type specimens. Currently, 38% of all geometrid BINs on BOLD have scientific species names, and 91% are identified at least to the level of genus.

## 4. Integration of field and museum: Papua New Guinea

DNA barcoding provides a framework for multiple lines of systematic and ecological research as part of a large-scale study of insect–plant ecology and biogeography in forests in Papua New Guinea (PNG) by the Binatang Research Centre [19]. The foundation of the programme is the characterization of the foliage-feeding insects reared from woody plants, but we are increasingly combining these data with that of light-trapped adults. DNA barcoding provides a rapid and accurate taxonomic framework, which is also instrumental in analysis of phylogeographic patterns [20], identifying caterpillars [21], detecting host–parasitoid interactions [22], linking historic types with modern specimens [23], describing new species [24] and phylogeny (including additional genes). DNA barcodes add great value to the taxonomic and ecological data, making them useful to a broad range of research enterprises, and allowing linkage with other projects including the global Geometridae inventory, especially by using BINs as surrogate species [18].

The PNG project, which started in 1994, was inspired by the ACG project referred to earlier, and shares significant overlap in approach, especially the application of parataxonomists as the core workforce and in then follow-up outsourcing specimens into the taxasphere. But we rapidly found that making identifications of individual species as they were encountered was inefficient, so we also undertook two initiatives to understand the PNG Lepidoptera fauna more systematically through museum collections. We undertook a digital atlas of Geometridae described from New Guinea, based on the type specimens, to which we added a DNA barcoding component [23,25]. We have also aggressively built a DNA barcode reference library from recent specimens in the Smithsonian collection from New Guinea, Borneo and the Pacific Islands (we currently have 19 000 voucher-based sequences of Lepidoptera from Papua New Guinea alone in BOLD). DNA barcodes serve for direct and more efficient integration of the data from field and museum collections by removing much of the dependence on scarce human resources in the taxasphere, just as has occurred in the ACG inventory.

## 5. Discussion

There is an obvious high relevance of a DNA library for taxonomy. It allows for large-scale screening for synonymies and for potential cryptic and new species, and is an integrated part of rapid species description pipelines [8,26,27] and thus considerably accelerates the alpha-taxonomic assessment of the global fauna [28–30]. Overcoming the ‘taxonomic impediment’ will be facilitated by:

(1) A new, comprehensive, solid basis for integrated taxonomy: the BOLD database provides each genetic cluster (usually corresponding to a species) with a BIN page, including high-quality images, georeferenced metadata and genetic data. Type specimens are included in this data pool, thereby pinning each genetic cluster to the scientific literature: for example, 3000+ geometrid-type barcodes have been added to date [29]. Data can easily be moved from BOLD to journals such as the *Biodiversity Data Journal* [31,32] and global databases (e.g. GenBank, GBIF). BOLD has furthermore proved to be an ideal platform for data sharing and international networking in taxonomy. Innumerable researchers—both professional and amateurs—have used BOLD data for the detection of cryptic species, for the analysis of relationships, and in the preparation of (i) alpha-taxonomic revisions, (ii) national/regional species inventories, and (iii) ecological analyses. The BIN system facilitates species delimitation, formation of species-groups and related uses in understanding speciation, evolution and scenarios of colonization in biogeography. The BIN system also provides a system of ‘interim names’ for species that is standardized and traceable on a global basis [18,33]. DNA barcodes are also being used to support large taxonomic monographs and regional assessments of the geometrid fauna of Europe [34] and Africa [35,36].
(2) Release time of taxonomists from routine identifications to do research and curation. These guild members usually spend much of their time answering questions and providing identification services in monitoring (e.g. impact studies and environmental surveys) and applied entomology (e.g. indoor pests, forestry and agriculture), often involving thousands of individuals of a few, usually common, species in our home countries. All of these services could be offered by a personal barcorder. Traditional Sanger sequencing as well as next-generation sequencing (NGS) methods can release experts from very time-consuming sorting and identification for the general public, and allow them (i) to establish the foundation for global biodiversity research and (ii) to be more active in capacity-building (including training of young scientists) and public outreach. Of course, the core problem is the lack of jobs for people not viewed as providing essential services for the paying public.
(3) Making taxonomy easier by reducing the need for travel to distant museums and helping museum curators to receive fewer loan requests and fewer visitors owing to the online-availability of images, collateral data and DNA barcodes of type specimens and other important vouchers in taxonomy.

**Figure 2.**
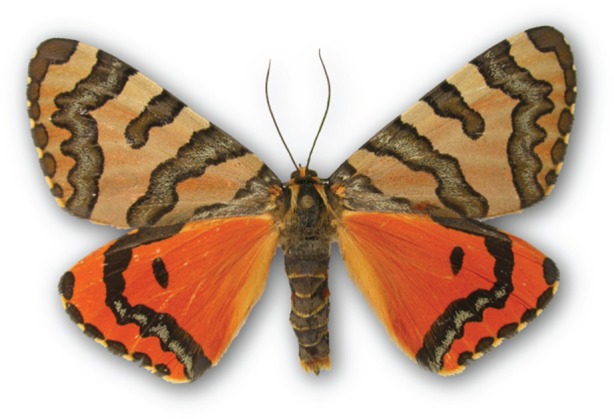
Paratype of *Callioratis mayeri* Staude, 2001, Long Hill, South Africa, DNA barcoded (BC ZSM Lep 00058) the sequence currently being used to delineate and describe a sister species from Drakensberge mountains in South Africa.

DNA barcodes of type specimens (figure 2) support alpha-taxonomy (i) by providing an objective and easily accessible reference to what a Linnean name is believed to represent and cross-referencing it to larger genetic clusters, (ii) by being non-destructive (e.g. a leg can be reassociated with the type specimen after DNA-extraction or use tissue obtained during genitalic dissections), and (iii) complementing other, new, non-invasive methods in morphology (e.g. microCT), thus opening new horizons for a modern standard data management for type specimens in museums. The success of geometrid DNA barcoding (as well as the BIO campaigns on Sphingidae and Saturniidae) and its prospects for taxonomy should encourage curators of natural history museums to join the initiative and to extend it to cover other families in a similar way. Type barcoding programmes should be initiated for all major museums and for all new descriptions, potentially introducing this as a recommendation or obligation for the Code of Zoological Nomenclature. BOLD records for type specimens can readily by linked to the original descriptions through the Biodiversity Heritage Library (www.Biodiversitylibrary.org), and GenBank has also enhanced its ability to handle information from type specimens [1]. All of these new tools can enhance traditional taxonomy, and become integrated taxonomy combining multiple character sets.

The Global Malaise Program [37] represents an innovative approach to scaling up DNA barcoding to produce voucher-based DNA barcodes cost-effectively for large samples. Rapidly evolving techniques for metabarcoding using NGS techniques will enhance biodiversity inventories by lowering per-sample costs, allowing analysis of environmental samples and allowing simultaneous identification of an organism and its food, parasites and endosymbionts [38–41]. But high-quality DNA barcode libraries linked to voucher specimens will remain important for providing identifications, and validating the links to the collateral data discussed above.

## 6. Conclusion

DNA barcoding is an important tool in biodiversity inventories, from facilitating workflows of identification of freshly collected specimens from the field, to describing the overall diversity of large taxa from museum collections, and most importantly linking the fresh specimens, the general museum collections and historic-type specimens. The process also flushes out unexpected sibling species hiding under long-applied scientific names, thereby clarifying and parsing previously mixed collateral data. BOLD has matured to an essential interactive platform for the multi-authored and multi-process collaboration. From our experience with the explosion of biodiversity database platforms in recent years, BOLD probably has the most impact in facilitating collaborations, not only among taxonomists, but across disciplines including ecology and biogeography. The BIN system and the related BOLD tools have become a powerful way around some parts of the ‘taxonomic impediment’, at least for entomology, by providing fast but testable and tractable species hypotheses, tools for visualizing the distribution of those in time and space, and an interim naming system for communication. These tools are all highly interoperable with related bioinformatics tools such as GenBank and the Biodiversity Heritage Library, and easily used by people in any language and with minimal formal education.
